# Crystal chemistry and compressibility of Fe_0.5_Mg_0.5_Al_0.5_Si_0.5_O_3_ and FeMg_0.5_Si_0.5_O_3_ silicate perovskites at pressures up to 95 GPa

**DOI:** 10.3389/fchem.2023.1258389

**Published:** 2023-10-06

**Authors:** Iuliia Koemets, Biao Wang, Egor Koemets, Takayuki Ishii, Zhaodong Liu, Catherine McCammon, Artem Chanyshev, Tomo Katsura, Michael Hanfland, Alexander Chumakov, Leonid Dubrovinsky

**Affiliations:** ^1^ Bayerisches Geo Institute (BGI), Universität Bayreuth, Bayreuth, Germany; ^2^ Department of Earth Sciences, University of Oxford, Oxford, United Kingdom; ^3^ Institute for Planetary Materials, Okayama University, Misasa, Japan; ^4^ State Key Laboratory of Superhard Materials, Jilin University, Changchun, China; ^5^ European Synchrotron Radiation Facility (ESRF), Grenoble, France

**Keywords:** bridgmanite, silicate perovskite, double perovskite, spin transition, single-crystal X-ray diffraction, synchrotron Mössbauer spectroscopy, high pressure

## Abstract

Silicate perovskite, with the mineral name bridgmanite, is the most abundant mineral in the Earth’s lower mantle. We investigated crystal structures and equations of state of two perovskite-type Fe^3+^-rich phases, FeMg_0.5_Si_0.5_O_3_ and Fe_0.5_Mg_0.5_Al_0.5_Si_0.5_O_3_, at high pressures, employing single-crystal X-ray diffraction and synchrotron Mössbauer spectroscopy. We solved their crystal structures at high pressures and found that the FeMg_0.5_Si_0.5_O_3_ phase adopts a novel monoclinic double-perovskite structure with the space group of *P21/n* at pressures above 12 GPa, whereas the Fe_0.5_Mg_0.5_Al_0.5_Si_0.5_O_3_ phase adopts an orthorhombic perovskite structure with the space group of *Pnma* at pressures above 8 GPa. The pressure induces an iron spin transition for Fe^3+^ in a (Fe_0.7_,Mg_0.3_)O_6_ octahedral site of the FeMg_0.5_Si_0.5_O_3_ phase at pressures higher than 40 GPa. No iron spin transition was observed for the Fe_0.5_Mg_0.5_Al_0.5_Si_0.5_O_3_ phase as all Fe^3+^ ions are located in bicapped prism sites, which have larger volumes than an octahedral site of (Al_0.5_,Si_0.5_)O_6_.

## 1 Introduction

**FIGURE 1:**
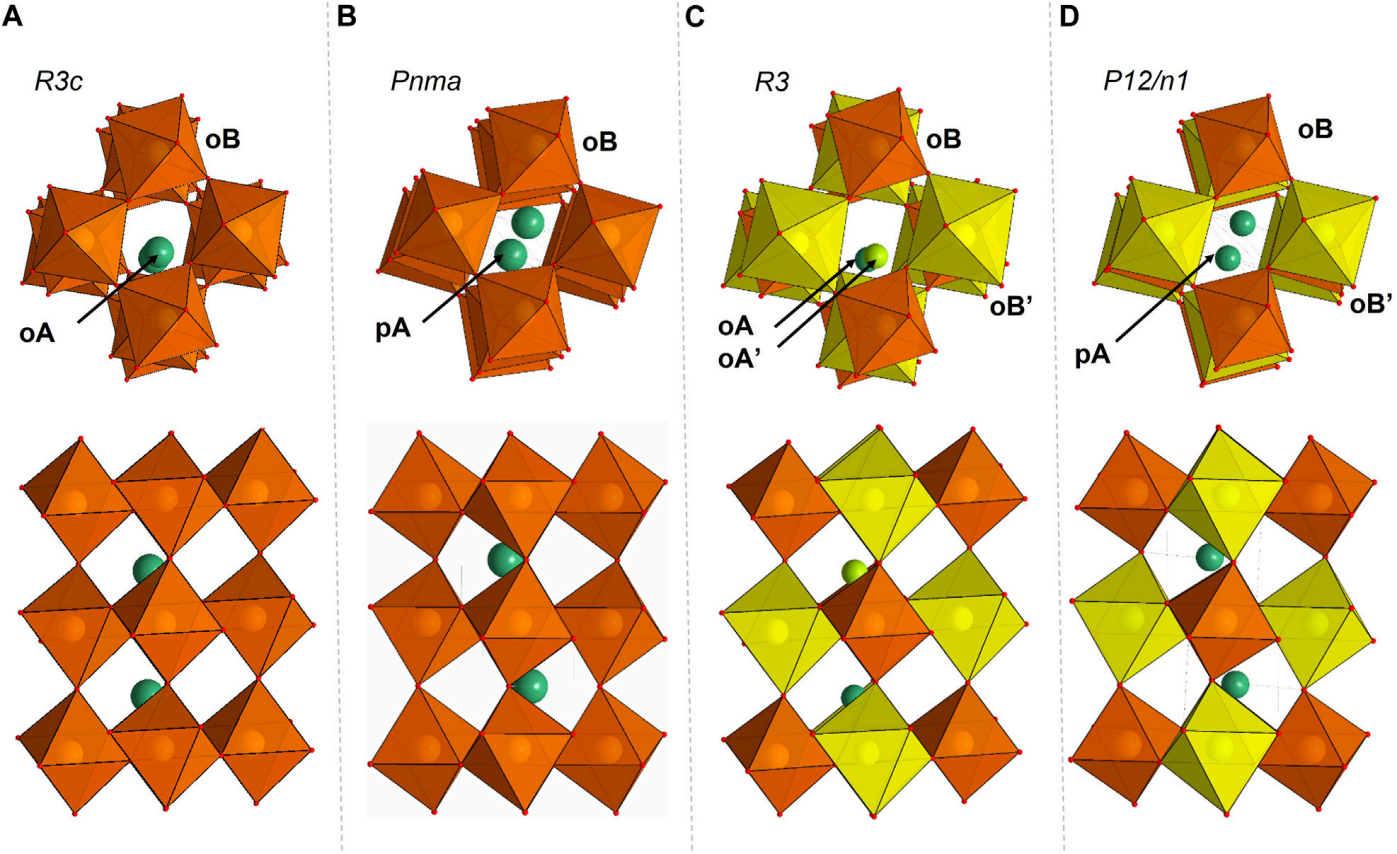
Crystal structures of Fe_0.5_Mg_0.5_Al_0.5_Si_0.5_O_3_ **(A,B)** and FeMg_0.5_Si_0.5_O_3_ **(C,D)** single crystals. The building blocks of the structures are octahedra and bicapped prisms. Cations and their surrounding polyhedra that correspond to the same crystallographic site are represented by the same color. **(A)** Fe_0.5_Mg_0.5_Al_0.5_Si_0.5_O_3_ adopts the LiNbO_3_-type structure with the space group of *R3c* at low pressures. In this structure, there are two octahedral crystallographic sites, notated as oA (green atoms, occupied by Fe_0.5_Mg_0.5_) and oB (orange octahedra, occupied by Al_0.5_Si_0.5_). oA and oB are connected through edges and faces. **(B)** Fe_0.5_Mg_0.5_Al_0.5_Si_0.5_O_3_ adopts the distorted perovskite structure with the space group of *Pnma* at high pressures. In this structure, there is one bicapped prism noted as pA (green atoms, occupied by Fe_0.5_Mg_0.5_) and one octahedral crystallographic site (orange octahedra, occupied by Al_0.5_Si_0.5_) noted as oB. All octahedra are connected through corners. Compared to its low-pressure structure, pA is formed from two oAs after phase transition. **(C)** FeMg_0.5_Si_0.5_O_3_ adopts the corundum derivative structure with the space group of *R3*. It consists of four different octahedral crystallographic sites, noted as oA (light green atom), oA’ (dark green atom), oB (yellow octahedra), and oB’ (orange octahedra). **(D)** FeMg_0.5_Si_0.5_O_3_ adopts the double-perovskite structure with the space group of *P12/n1*. It consists of one bicapped prism crystallographic site, noted as pA (dark green atom), and two octahedral crystallographic sites, noted as oB (orange octahedra, occupied by Si only) and oB’ (yellow octahedra).

The most abundant mineral on the Earth, magnesium silicate perovskite (bridgmanite), crystallizes in an orthorhombic GdFeO_3_-type perovskite structure and consists of large distorted “bicapped prism” sites (pA-sites) in the voids of the three-dimensional net of corner-sharing octahedra (oB-sites) (Figure 1C). Compressibility (Fiquet et al., 2000; Tsuchiya et al., 2004; Vanpeteghem et al., 2006) and Brillouin spectroscopy (Sinogeikin et al., 2004) studies on the MgSiO_3_ bridgmanite end-member reported isothermal bulk modulus values ranging between 259 and 268 GPa. The effect of Al and Fe content on bridgmanite compressibility remains unclear due to limited information on the substitution mechanisms, iron oxidation state (Mao et al., 1991; Kubo et al., 2000; Andrault et al., 2001; Nishiyama et al., 2007), and cation distribution in the samples (Saikia et al., 2009). The Fe^3+^ content may be significant in bridgmanite even at low oxygen fugacity (*f*O_2_) (Frost et al., 2004), and Fe^3+^ may cause significant changes in the elastic properties of the material (Saikia et al., 2009; Ballaran et al., 2012). Moreover, the presence of oxygen vacancies strongly decreases the bulk modulus (Ismailova et al., 2016); therefore, substitution mechanisms occurring during sample synthesis should be considered (Mao et al., 2015; Shukla and Wentzcovitch, 2016).

Fe- and Al-rich sample syntheses for further diamond anvil cell (DAC) experiments are challenging because it is difficult to obtain homogeneous compositions with sufficiently large crystals (up to micrometers) of a quality suitable for single-crystal X-ray diffraction (SC-XRD). Therefore, previous X-ray diffraction experiments at high pressures were usually limited by low Fe and Al contents (Ballaran et al., 2012; Glazyrin et al., 2014) in the sample or with the use of powder diffraction, leading to complicated data interpretation for the studies of silicate crystal chemistry at high pressure (Liu et al., 2018; Zhu et al., 2020).

In the present study, we synthesized high-quality crystals of high-pressure silicates with high Fe and Al contents, employing a multi-anvil apparatus. The samples were further loaded in diamond anvil cells for *in situ* SC-XRD experiments up to 60 GPa and the Mössbauer spectroscopy study at pressures up to 95 GPa. We unambiguously identified the structure of high-pressure Fe-bearing Al-free silicate FeMg_0.5_Si_0.5_O_3_ as double perovskites with two octahedral sites, one (oB) occupied by silicon and another (oB’) by ferric iron and magnesium. We were able to observe the volume collapse of the Fe^3+^-bearing oB’-site and changes in Mössbauer parameters at pressures above 40 GPa, which were previously associated with the spin transition. We were also able to derive the “FeAlO_3_” end-member bulk modulus. Our results show that compositional variations in bridgmanite have an impact on the structure and crystal chemistry and lead to the appearance of a more complex and unusual phase, silicate double perovskites.

## 2 Materials and methods

### 2.1 Sample synthesis and characterization

Fe_0.5_Mg_0.5_Al_0.5_Si_0.5_O_3_ and FeMg_0.5_Si_0.5_O_3_ single crystals were synthesized using the Kawai-type multi-anvil press with the Osugi-type module (Ishii et al., 2019) at Bayerisches Geoinstitut, IRIS-15 (Ishii et al., 2016). A detailed description of the sample synthesis procedure can be found in Liu et al. (2019). The chemical composition of the recovered samples was determined using a JEOL JXA-8200 Electron Probe Microanalyzer (EPMA). The oxidation state of iron was determined by Mössbauer spectroscopy. Within the detection limits of the measurements, all the iron in FeMg_0.5_Si_0.5_O_3_ is represented as Fe^3+^. Approximately 16(4)% of iron in Fe_0.5_Mg_0.5_Al_0.5_Si_0.5_O_3_ is represented as Fe^2+^ (Supplementary Figure S1).

### 2.2 High-pressure experiments

BX90-type (Kantor et al., 2013) diamond anvil cells with diamond culet sizes ranging from 120 to 250 μm were used for conducting high-pressure experiments. To create a sample chamber between diamonds, rhenium gaskets were pre-indented to a thickness of 30 ± 5 μm. Subsequently, a laser was used to drill a hole in the center of the indented area, creating a sample chamber with a diameter of 50–110 μm depending on diamond culet sizes. Pre-selected single crystals were loaded into the center of the sample chamber together with a ruby sphere for pressure determination (Mao et al., 1986) at low pressures (*p* <6 GPa). Neon gas was loaded (Kurnosov et al., 2008) around the samples to serve as pressure-transmitting mediums, minimizing the degree of deviatoric stress. Additionally, it was also used for pressure determination at high pressures (*p* >6 GPa; Fei et al., 2007).

### 2.3 Single-crystal X-ray diffraction

SC-XRD patterns were collected at the ID15B beamline at the European Synchrotron Radiation Facility (ESRF). An X-ray beam with the energy of 30 keV (*λ* = 0.4133 Å) was used, and diffraction data were collected using a MAR555 flat-panel detector. At each pressure point, SC-XRD data collection was performed in the omega range of ± 38° or ± 32°, depending on the DAC opening angle, with a 0.5° step and exposure time of 1 s for each step. The integration of the reflection intensities and absorption corrections was performed using CrysAlis^Pro^ (Agilent, 2014). The structure solution and refinement were performed in the isotropic approximation using Jana2006 (Petrícek et al., 2014) with Superflip (Palatinus and Chapuis, 2007) and SHELXT (Sheldrick, 2015).

### 2.4 Synchrotron Mössbauer spectroscopy

Energy-domain synchrotron Mössbauer spectroscopy measurements were carried out at the nuclear resonance beamline ID18 at ESRF (Rüffer and Chumakov, 1996), using the synchrotron Mössbauer source (Potapkin et al., 2012). The spot size of the focused beam was approximately 15 μm^2^ × 15 μm^2^. Due to the usage of ^57^Fe in the starting material during sample synthesis, spectral acquisition times were less than 1 h. Therefore, we do not expect an appearance of spectral features associated with the signal obtained from Fe contained in the Be window and lenses, as stated in the previous studies.

## 3 Results and discussion

### 3.1 Crystal structures of Fe_0.5_Mg_0.5_Al_0.5_Si_0.5_O_3_

At ambient conditions, Fe_0.5_Mg_0.5_Al_0.5_Si_0.5_O_3_ adopts a LiNbO_3_-type structure (space group *R3c*; Figure 1). The lattice parameters are determined to be *a* = *b* = 4.8790(1) Å and *c* = 12.9112(1) Å. This structure consists of two types of octahedra, oA and oB, forming corundum-like layers stacked along the crystallographic *c* axis (Ishii et al., 2017). Octahedral oA-sites are occupied by Fe and Mg (with an atomic ratio of 1:1), and oB-sites are occupied by Al and Si (with an atomic ratio of 1:1). On compression, Fe_0.5_Mg_0.5_Al_0.5_Si_0.5_O_3_ is stable in the LiNbO_3_-type structure up to 8 ± 2 GPa (Figure 4), above which it transforms into a distorted perovskite structure (space group *Pnma*; Figures 1B, 4). As expected from the difference in ionic radii in the octahedral coordination (Shannon, 1976), the volume of oA octahedra is larger than that of oB (Figure 5). The phase transitions occur through the tilt of oB octahedra and a shift of oA-site cations to form eight-fold prismatic sites (pA in Figures 1A, C).

### 3.2 Crystal structures of FeMg_0.5_Si_0.5_O_3_

Compared to Fe_0.5_Mg_0.5_Al_0.5_Si_0.5_O_3_, FeMg_0.5_Si_0.5_O_3_ exhibits a more complex structure, as supported by the systematic absence analysis (Supplementary Figure S2). At ambient conditions, it adopts a corundum derivative structure (space group *R3*; Figure 1C), with the lattice parameters determined to be a = b = 4.9406(7) Å and c = 13.319(2) Å. Two octahedra, labeled as oA and oA’, are occupied by Fe and Mg, with site occupancies of (Fe_0.6_Mg_0.4_) and (Fe_0.7_Mg_0.3_), respectively, and are located in every second layer along the *c*-direction. Another two octahedra, oB and oB’, are occupied by Si and (Mg_0.7_Fe_0.3_). The difference in oB- and oB’-site occupancies causes the difference in octahedra volumes at ambient conditions: oB has a volume of 8 Å^3^, while oB’ has a volume of 10.4 Å^3^. It is worth noting that oB and oB’ are located in layers along *c*-directions and do not mix with the layers of oA and oA’ octahedra. It is a new structure, which has not been observed previously for corundum derivatives. Upon compression, FeMg_0.5_Si_0.5_O_3_ is stable in the corundum derivative structure up to 12 ± 2 GPa, above which it transforms into a double-perovskite structure (space group *P12/n1*; Figures 1D, 4). This structure has some unique features that were not observed in other compositions previously. First, we observed the ordering of cations located on octahedral sites (oB and oB’), which leads to a symmetry decrease from orthorhombic perovskites to monoclinic double perovskites (Figure 1D). As a result, two different oB- and oB’-sites remain distinguishable after phase transition at 12 GPa (Figure 5). Second, we see that a larger pA-site is occupied by Mg and Fe based on single-crystal X-ray diffraction data refinement. The average pA-site Fe^3+^ occupancy is approximately 0.62(3) and is kept for all measured pressure points (Supplementary Figure S3). Another oB-site is occupied only by Si, while the oB’-site contains Fe and sufficient amounts of Mg (∼30%). The derived cation distribution is not an artifact because an attempt to refine structures within the oB-site occupied by both Si and Fe, or oB’ without the Mg-worth refinement quality (R_all_ increase; see Supplementary Figure S4 for more information). Considering all the available data, we conclude that, in all previously investigated compositions, the pA-site is occupied by Fe_0.6_Mg_0.4_ and the oB’-site is occupied by Fe_0.7_Mg_0.3_. More information on structure refinement can be found in Supplementary Tables S1–S3.

### 3.3 Iron: oxidation and spin states

**FIGURE 2:**
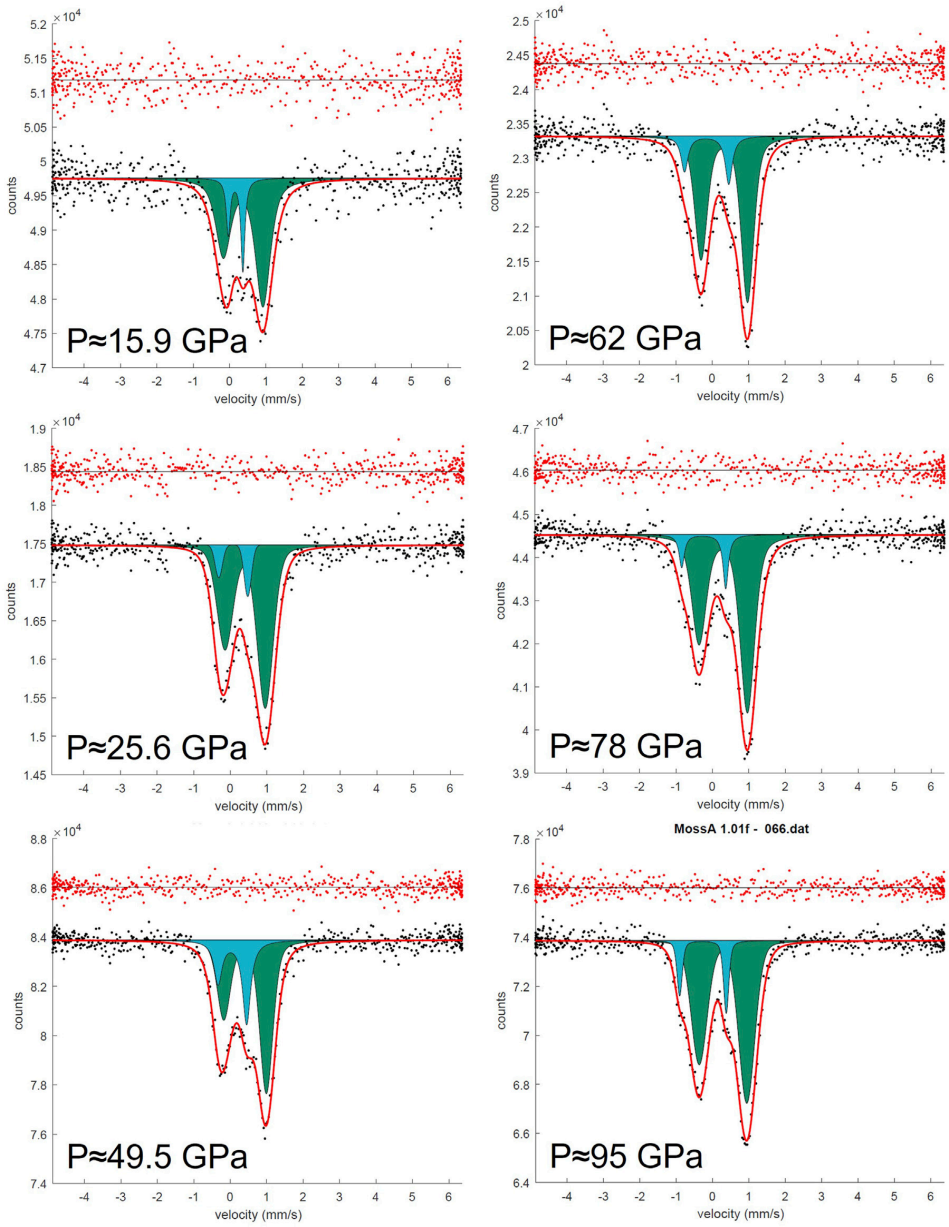
SMS spectra of the FeMg_0.5_Si_0.5_O_3_ sample during the pressure increase from 15.9 to 95 GPa in DAC. All spectra were collected on ID18, ESRF. Doublets that correspond to Fe^3+^ cations located on the pA-site are represented in green, and doublets that correspond to Fe^3+^ located on the oB’-site are represented in blue.

**FIGURE 3:**
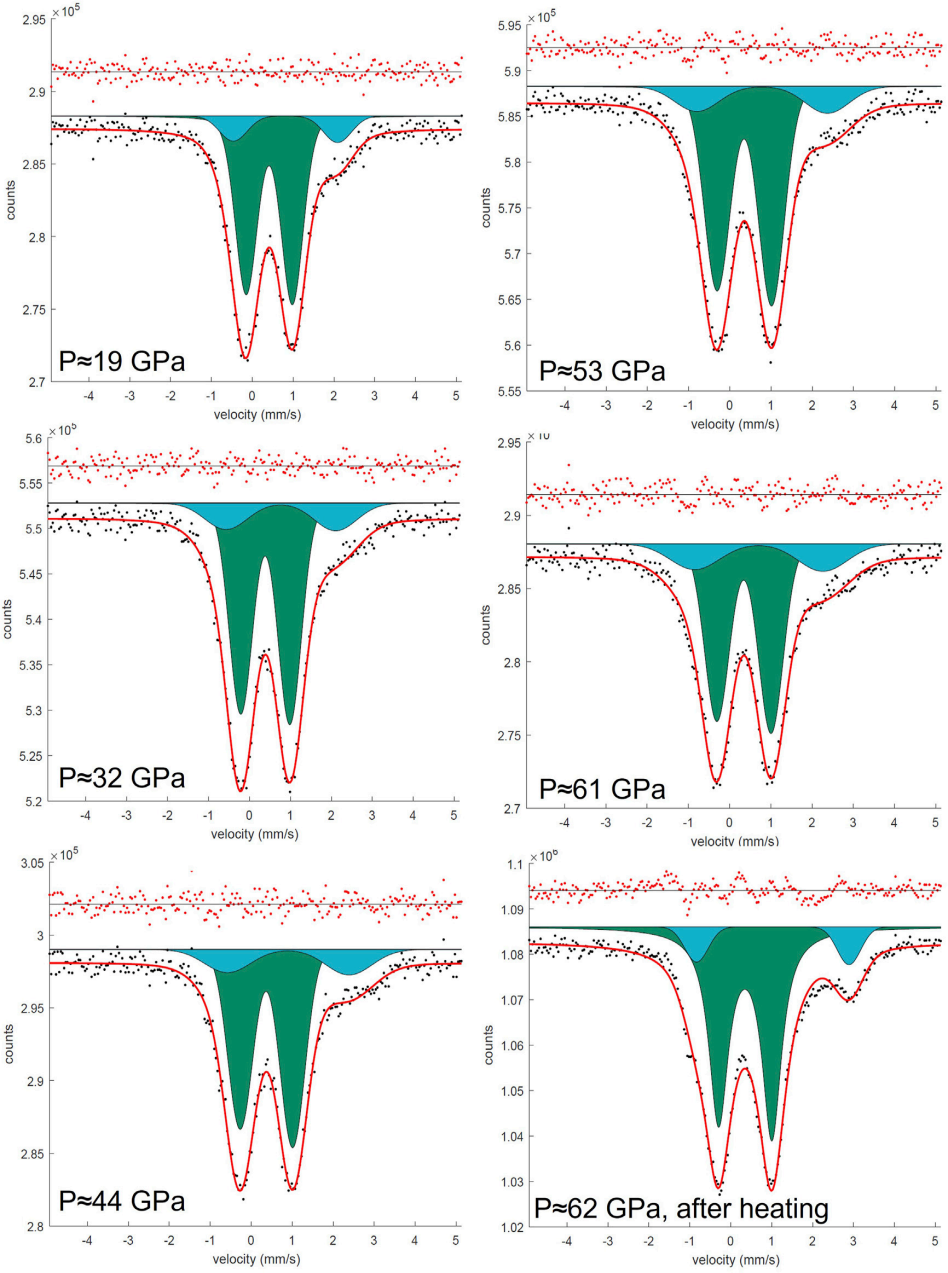
SMS spectra of the Fe_0.5_Mg_0.5_Al_0.5_Si_0.5_ sample during the pressure increase from 19 to 62 GPa in DAC. All spectra were collected on ID18, ESRF. Doublets that correspond to Fe^3+^ cations located on the pA-site are represented in green, and doublets that correspond to Fe^3+^ located on the oB’-site are represented in blue.

Synchrotron Mössbauer spectra were collected up to 95 GPa for FeMg_0.5_Si_0.5_O_3_ (Figure 2) and up to 62 GPa for Fe_0.5_Mg_0.5_Al_0.5_Si_0.5_O_3_ (Figure 3). The Fe_0.5_Mg_0.5_Al_0.5_Si_0.5_O_3_ sample contains noticeable amounts of ferrous iron. Due to the broadness of the doublet attributed to pA-site Fe^2+^, the precise determination of Fe^3+^ content at ambient conditions is difficult. However, from the Mössbauer spectra collected at high pressure, we can estimate the Fe^2+^ content to be approximately 16(4)%. This suggests the presence of less than 1% oxygen vacancies, which, however, cannot be detected directly from our microprobe data (uncertainty of the oxygen content is ∼3%). Mössbauer spectroscopy at ambient conditions and at high pressures shows that all iron in FeMg_0.5_Si_0.5_O_3_ is Fe^3+^ (Supplementary Figure S1). Upon compression, Fe^3+^ in Fe_0.5_Mg_0.5_Al_0.5_Si_0.5_O_3_ does not undergo spin transition (Figure 6).

The near absence of Fe^2+^ in FeMg_0.5_Si_0.5_O_3_ together with the presence of high amounts of Fe^3+^ on oB-sites allowed us to unambiguously distinguish two doublets on Mössbauer spectra, which correspond to Fe^3+^ located on pA- and oB’-crystallographic sites. Even at pressures lower than the expected range for a spin crossover, one could still clearly observe the doublet for Fe^3+^ on oB’-sites (Figure 2). We show that the “New component” that appears in Sinmyo et al. (2017), and which was assigned to low spin Fe^3+^, existed at pressures before the spin crossover but was difficult to observe because of strong overlapping with intense A-site Fe^2+^ and Fe^3+^ doublets. Other evidence for this is the decrease in the Fe^3+^ doublet relative area, as reported by Sinmyo et al. (2017). Furthermore, hyperfine parameters that we associate with Fe^3+^ on oB-sites in FeMg_0.5_Si_0.5_O_3_ double perovskites are similar to those for a non-magnetic doublet, reported by Kupenko et al., 2019, collected on ζ-Fe_2_O_3_, which has a strongly distorted perovskite-like structure (Bykova et al., 2013; Bykova et al., 2016). Our results are generally consistent with a result on the sample with a composition similar to FeMg_0.5_Si_0.5_O_3_ from Liu et al. (2018), where they reported the central shift (CS) between the high-spin and low-spin states in the order of 0.2 mm/s based on the nuclear forward scattering experiment, which, however, does not allow to unambiguously determine the actual CS values. In agreement with the previous studies, we observe the QS increase with pressure for both studied compositions (Figure 6) (Sinmyo et al., 2017; Xiao et al., 2017; Supplementary Table S4). For example, doublets that correspond to Fe^2+^ on the pA-site in Fe_0.5_Mg_0.5_Al_0.5_Si_0.5_O_3_ perovskites have QS values of 2.50(5) mm/s at 19 GPa and 3.73(5) mm/s at 62 GPa (Figure 6).

### 3.4 Compressibility and effect of Al^3+^ and Fe^3+^ on the volume of the octahedra

**FIGURE 4:**
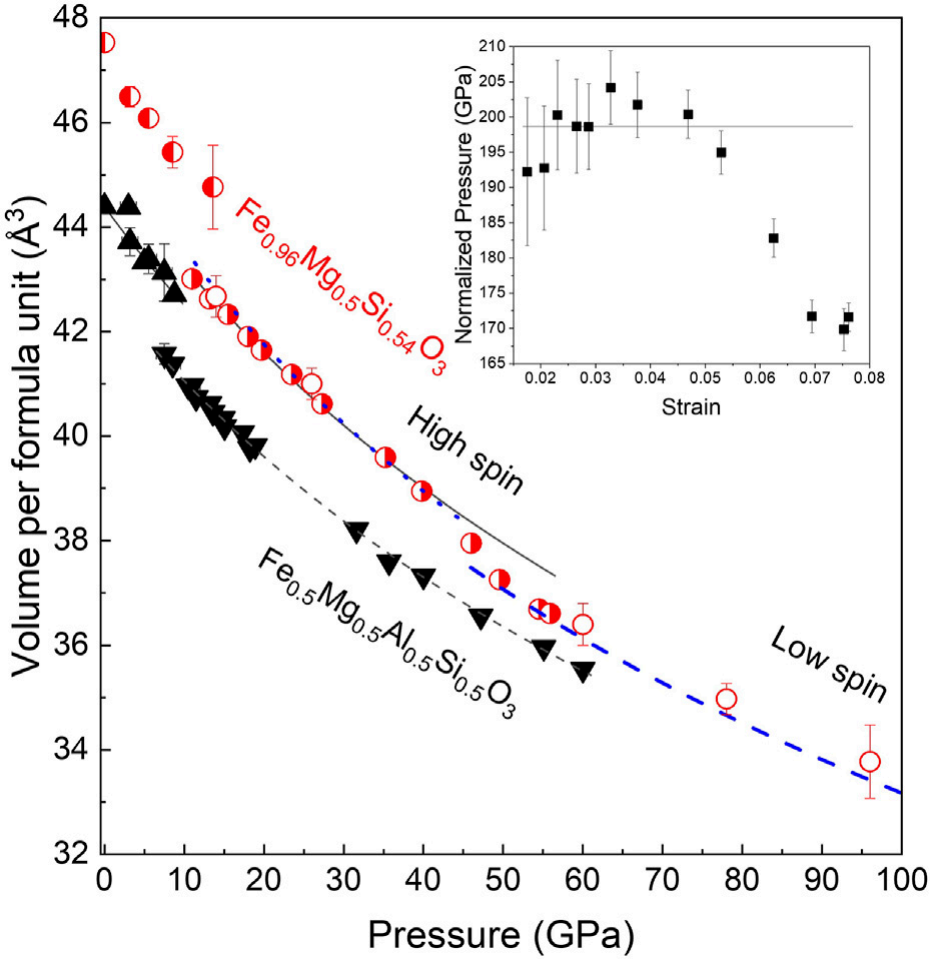
Volume per formula unit of Fe_0.5_Mg_0.5_Si_0.5_Al_0.5_O_3_ (black triangles) and FeMg_0.5_Si_0.5_O_3_ (red circles) as a function of pressure at room temperature. For FeMg_0.5_Si_0.5_O_3_, the three open circles at the pressure above 60 GPa correspond to data points that show that single-crystal X-ray diffraction (SC-XRD) refinement was not possible due to poor data quality. The lines represent fittings of pressure–volume data with the second-order Birch–Murnaghan equation of state (BM2–EoS) for different structures and compositions. Fe_0.5_Mg_0.5_Si_0.5_Al_0.5_O_3_ is stable in the LiNbO_3_-type structure below 8 ± 2 GPa. The BM2–EoS fitting gives K_0_ = 211 ± 10 GPa and V_0_ = 266.35 ± 0.08 Å^3^. Above 8 ± 2 GPa, Fe_0.5_Mg_0.5_Si_0.5_Al_0.5_O_3_ adopts a distorted perovskite structure with a smaller volume but a higher bulk modulus (V_0_ = 171.2 ± 0.2 Å^3^; K_0_ = 221 ± 3 GPa). FeMg_0.5_Si_0.5_O_3_ is stable in the trigonal corundum derivative structure below 12 ± 2 GPa. Above 12 ± 2 GPa, it adopts a monoclinic double-perovskite structure. The BM2–EoS fitting of data between 12 and 40 GPa gives K_0_ = 199 ± 6 GPa and V_0_ = 181.2 ± 0.5 Å^3^. Above 40 GPa, the elastic softening of FeMg_0.5_Si_0.5_O_3_ is related to the spin transition of Fe^3+^ located in the B-sites of perovskite. Inset: normalized pressure as a function of the Eulerian strain for FeMg_0.5_Si_0.5_O_3_ shows the applicability of the use of BM2–EoS and marks the discontinuity between data points at pressures higher than 40 GPa.

The volume per formula unit, as a function of pressure at an ambient temperature, is shown for the two samples in Figure 4. Discontinuities corresponding to the phase transition from the LiNbO_3_-type structure to a perovskite structure in the case of Fe_0.5_Mg_0.5_Al_0.5_Si_0.5_O_3_ and to phase transition from a new corundum-related structure to the double-perovskite structure in the case of FeMg_0.5_Si_0.5_O_3_ are observed at 8 ± 2 and 12 ± 2 GPa, respectively. The fitting of pressure–volume data for perovskite-structured phases with the second-order Birch–Murnaghan equation of state (BM2–EoS) resulted in a bulk modulus of 212(3) GPa for Fe_0.5_Mg_0.5_Al_0.5_Si_0.5_O_3_ and a bulk modulus of 199(6) GPa for FeMg_0.5_Si_0.5_O_3_. The bulk moduli of the two perovskites are smaller than that of the MgSiO_3_ perovskite (251 GPa, Ballaran et al., 2012).

**FIGURE 5:**
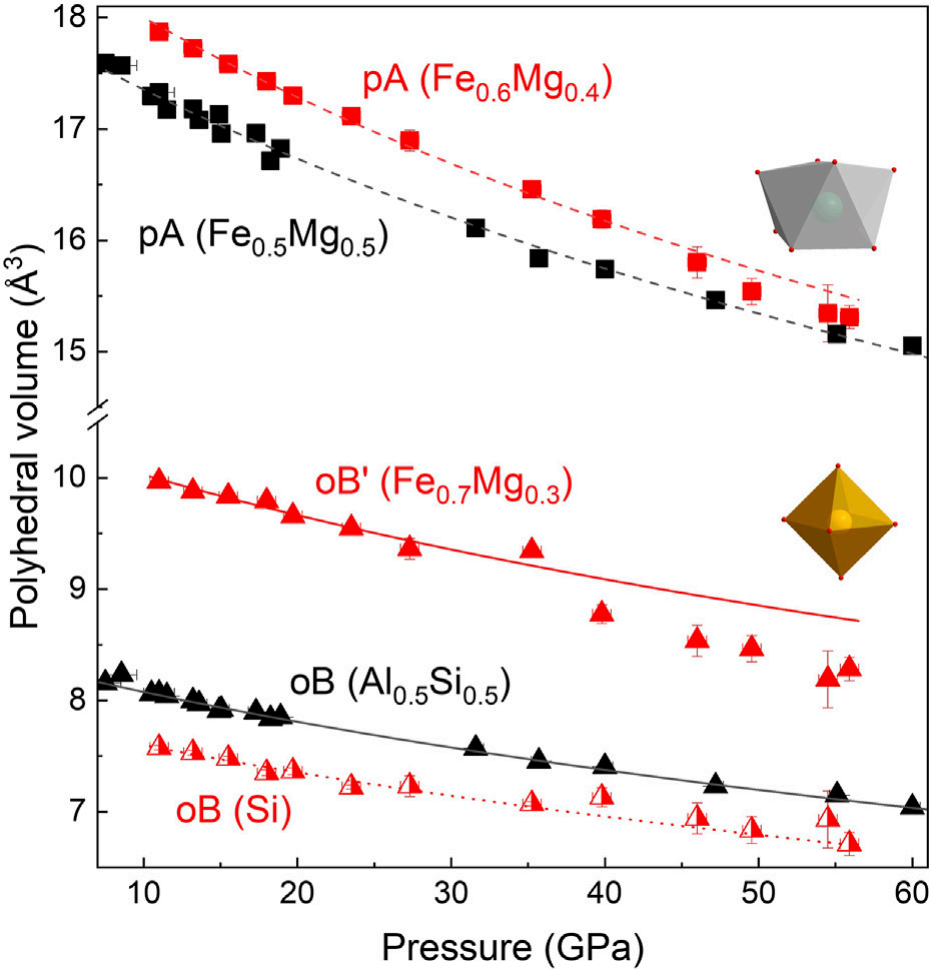
Compression behavior of polyhedra for Fe_0.5_Mg_0.5_Al_0.5_Si_0.5_O_3_ (black triangles) and FeMg_0.5_Si_0.5_O_3_ (red triangles). The volume of the bicapped prism is shown as squares. The volume of octahedra (oB and oB’) is shown as triangles. For Fe_0.5_Mg_0.5_Al_0.5_Si_0.5_O_3_, pA- and oB-sites have a smooth volume decrease during compression, and A-sites (K_A0_ = 216 ± 6 GPa) are more compressible than B-sites (K_B0_ = 238 ± 7). For FeMg_0.5_Si_0.5_O_3_, oB and oB’ are clearly distinguishable by volume as Si-bearing oB’-sites are smaller in volume. The change in the compressional behavior of the oB’-site above 40 GPa indicates the spin crossover of Fe^3+^.

In the case of FeMg_0.5_Si_0.5_O_3_, we observed an increase in compressibility at pressures above 40 GPa, which is further supported by stress versus the Eulerian strain plot from the inset of Figure 4. The same observation was made by Liu et al. (2018) on a sample with a similar composition. The high-pressure single-crystal X-ray diffraction data allowed us to track individual polyhedral volumes of the studied materials with increasing pressure. Although there is no change in the compressional behavior of pA- and oB-sites in the Al-rich sample, our experimental results clearly demonstrate the softening of Fe^3+^-bearing B’ octahedra in the FeMg_0.5_Si_0.54_O_3_ sample (Figure 5). There is a very weak tendency in the volume increase of SiO_6_ (B-site) octahedra with pressure across the spin crossover in oB’. A similar effect was observed in siderite (i.e., decrease in the volume of Fe^2+^O_6_ octahedra due to the spin transition and increase in C–O distances in CO_3_ groups (Lavina et al., 2010)).

**FIGURE 6:**
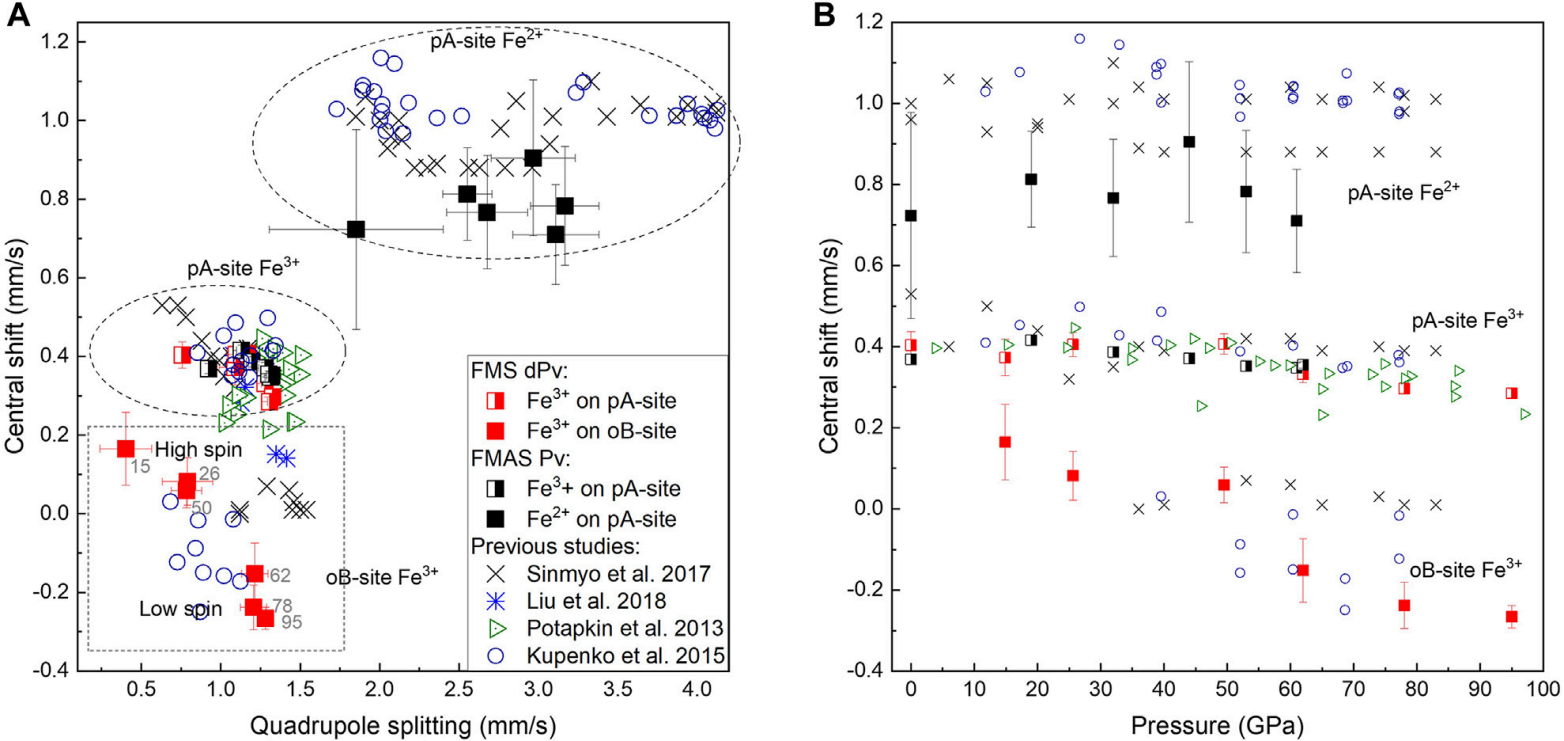
Central shift as a function of quadrupole splitting **(A)** and pressure **(B)**. Data points from this study are shown as squares (black symbols for Fe_0.5_Mg_0.5_Al_0.5_Si_0.5_O_3_ and red symbols for FeMg_0.5_Si_0.5_O_3_). Doublets related to Fe^3+^ on pA-sites are colored in half. Liu et al., 2018 (blue stars) used nuclear forward scattering (discussed in the main text), Sinmyo et al., 2017 (black crosses), and Potapkin et al., 2013 (green triangles) used synchrotron Mössbauer spectroscopy (SMS). The values near red squares correspond to the pressure (in GPa) at which the SMS data was collected.

**FIGURE 7:**
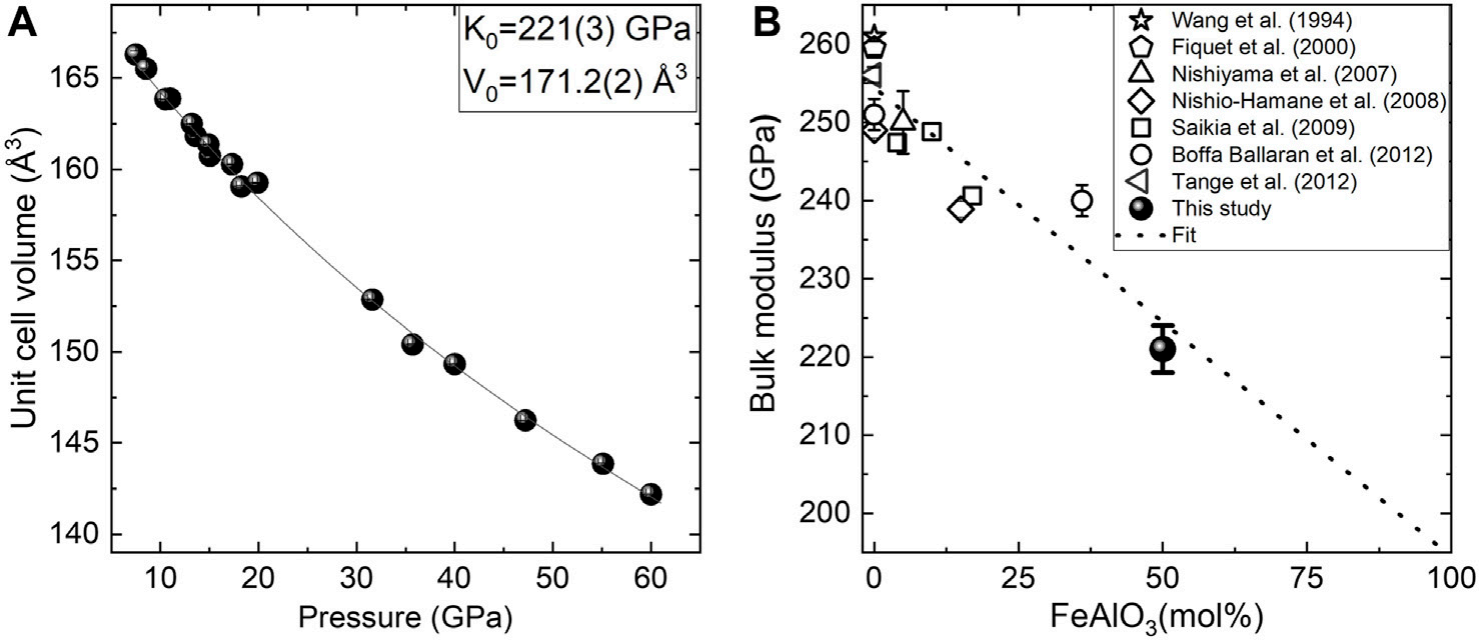
**(A)** Unit cell volume versus the pressure of Fe_0.5_Mg_0.5_Si_0.5_Al_0.5_O_3_ bridgmanite. Experimental points obtained in different experimental runs and fitted with the second-order Birch–Murnaghan equation of state (continues line). **(B)** Bridgmanite bulk modulus of Fe and Al-bearing bridgmanites as the function of the FeAlO_3_ content in the solid solution. Linear fitting results in K_0_ = 194(8) for end-member FeAlO_3_.

Our data indicate that in the case of Fe_0.5_Mg_0.5_Al_0.5_Si_0.5_O_3_ perovskites, a pA-site is more compressible than oB, as it was suggested previously (Sinmyo et al., 2017). When our result is considered together with previous data on the bulk compressibility of Fe-rich perovskites (Saikia et al., 2009; Ballaran et al., 2012; Dorfman et al., 2012; Glazyrin et al., 2014; Ismailova et al., 2016; Liu et al., 2018), one could see that it follows the general trend for the bulk modulus decrease with an increase in the Fe^3+^ content (Supplementary Figure S5). Moreover, the calculation of the bulk modulus for the Al-rich sample together with previously reported bulk moduli for Al, the Fe-bearing bridgmanite, allows us to constrain the bulk modulus of the pure FeAlO_3_ end-member K_0_ = 194 ± 8 GPa (Figure 7). Potentially, one could estimate the composition from polyhedra volumes; however, this result might be affected by the uncertainty of Fe spin and oxidation states.

**FIGURE 8:**
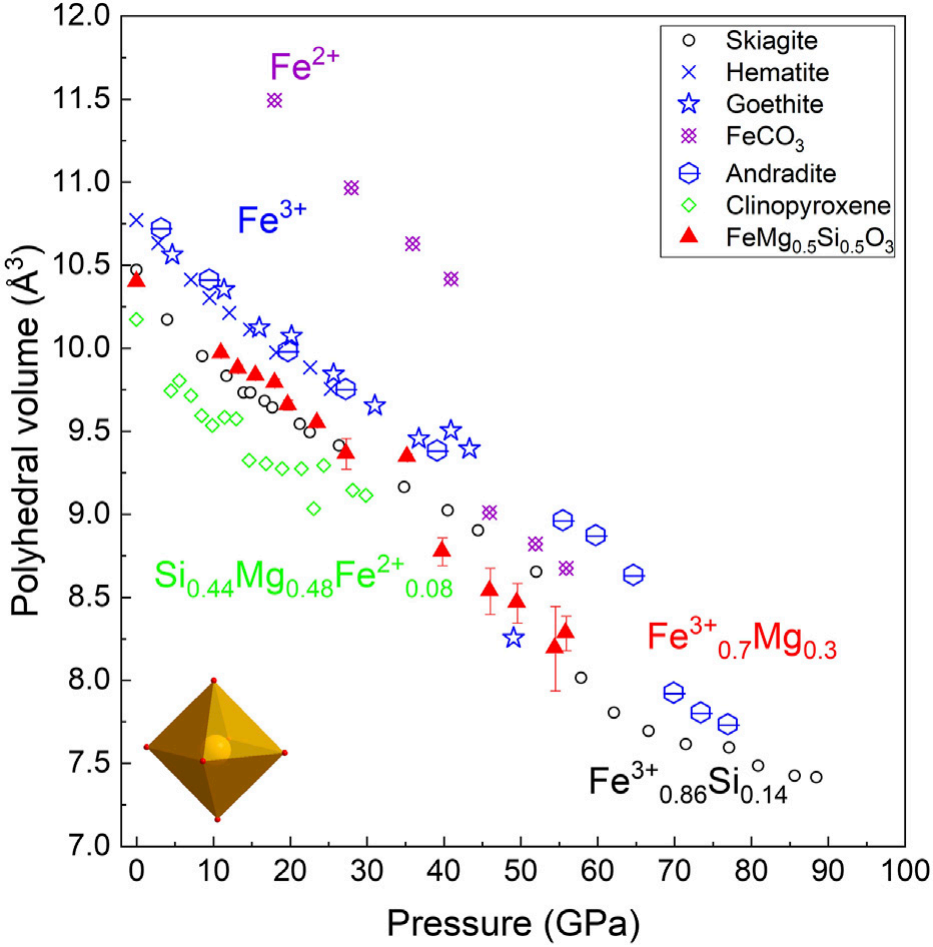
(Fe^3+^_0.7_ and Mg_0.3_) octahedra compression behavior in FeMg_0.5_Si_0.5_O_3_ (this study, red triangles) in comparison with skiagite, hematite, goethite, iron carbonate, andradite, and clinopyroxene, according to the previous studies and references therein (Vasiukov et al., 2017).

Although it is generally assumed that the volume collapse of Fe^3+^-rich bridgmanite at pressures above 40 GPa is associated with the spin transition, our new data on the Fe^3+^-bearing octahedra volume remain controversial. Figure 8 shows the oB’ volumes of the FeMg_0.5_Si_0.5_O_3_ sample (Mg and Fe^3+^-bearing octahedra) at all pressures are lower than that reported for pure Fe^3+^ octahedra in andradite, goethite, and hematite, despite the presence of Mg on the oB’-site suggesting a volume increase compared to pure Fe^3+^ octahedra. The low spin state of Fe^3+^ at ambient pressures was previously reported for various metal–organic compounds (Nihei et al., 2007), so one of the possible explanations for the observed effect is the low spin state of octahedral Fe^3+^ at low pressure. Indeed, the estimated ionic radii of Fe^3+^_0.7_Mg_0.3_, assuming a low spin state of ferric iron, is lower than that for the high spin state of Fe^3+^.

## 4 Conclusion

We investigated the high-pressure crystal chemistry of well-characterized crystalline materials with two compositions, FeMg_0.5_Si_0.5_O_3_ and Fe_0.5_Mg_0.5_Al_0.5_Si_0.5_O_3_, synthesized by the Kawai-type multi-anvil apparatus. We performed a series of compressibility experiments in diamond anvil cells up to 95 GPa. During compression, we collected single-crystal X-ray diffraction patterns and Mössbauer spectra, which allowed us to follow up on the changes in the crystal chemistry and Fe spin state during compression.

Fe_0.5_Mg_0.5_Al_0.5_Si_0.5_O_3_ with the LiNbO_3_-type structure at ambient conditions transforms into the perovskite-type phase at 8 GPa, and no spin transition was observed. On the other hand, FeMg_0.5_Si_0.5_O_3_ has a novel structure at ambient conditions: the low-pressure phase is the corundum-related type (space group *R3*), and at above approximately 12 GPa, it transforms into a new silicate double perovskite. An outstanding feature of the silicate double perovskite structure is having two individual octahedral sites: one occupied by Si only, and the other by iron and magnesium. Single-crystal X-ray diffraction Mössbauer spectroscopy data showed the compressibility changes of individual polyhedra and the variation in Mössbauer hyperfine parameters.

## Data Availability

The original contributions presented in the study are included in the article/Supplementary Material; structural data is deposited in the CCDC database repository (https://www.ccdc.cam.ac.uk/structures/), accession numbers 2294965, 2294966, 2294967, 2294968. Further inquiries can be directed to the corresponding authors.
