# Supplementary material for: Crystal chemistry and compressibility of Fe0.5Mg0.5Al0.5Si0.5O3 and FeMg0.5Si0.5O3 silicate perovskites at pressures up to 95 GPa
Source: Front Chem. 2023 Oct 6;11:1258389. doi: 10.3389/fchem.2023.1258389 (PMC10587407; doi:10.3389/fchem.2023.1258389)
Supplement: Supplementary file 1 [file DataSheet1.pdf]

Supplementary Material

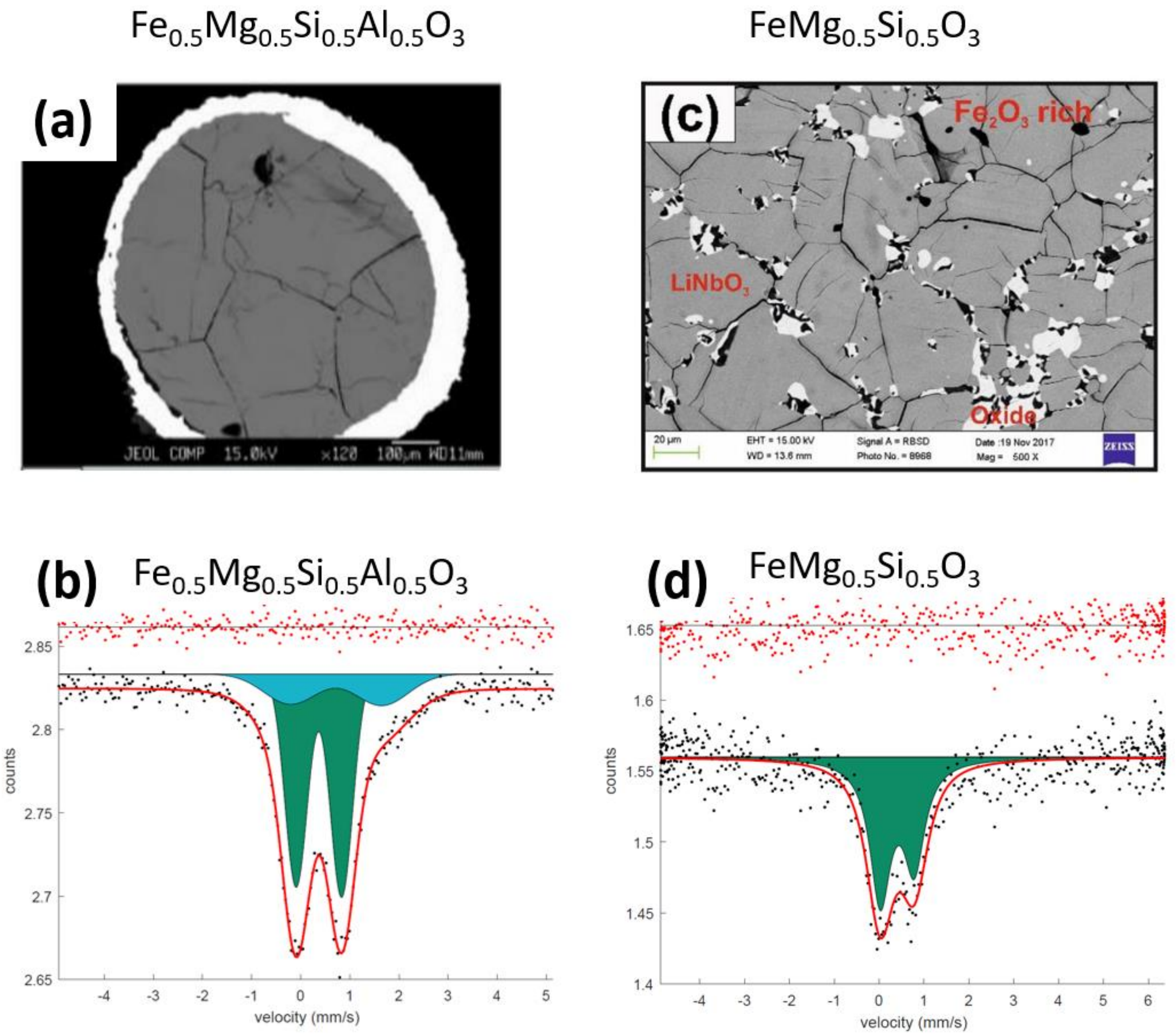

Supplementary Figure 1. Back-scattered electron images of samples recovered after multi-anvil experiments for a) $Fe_{0.5}Mg_{0.5}Si_{0.5}Al_{0.5}O_3$ c) $FeMg_{0.5}Si_{0.5}O_3$ and Mössbauer spectra of recovered corundum-related phase at ambient conditions for b) $Fe_{0.5}Mg_{0.5}Si_{0.5}Al_{0.5}O_3$ d) $FeMg_{0.5}Si_{0.5}O_3$. The blue doublet corresponds to Fe^{2+} and green one to Fe^{3+}.

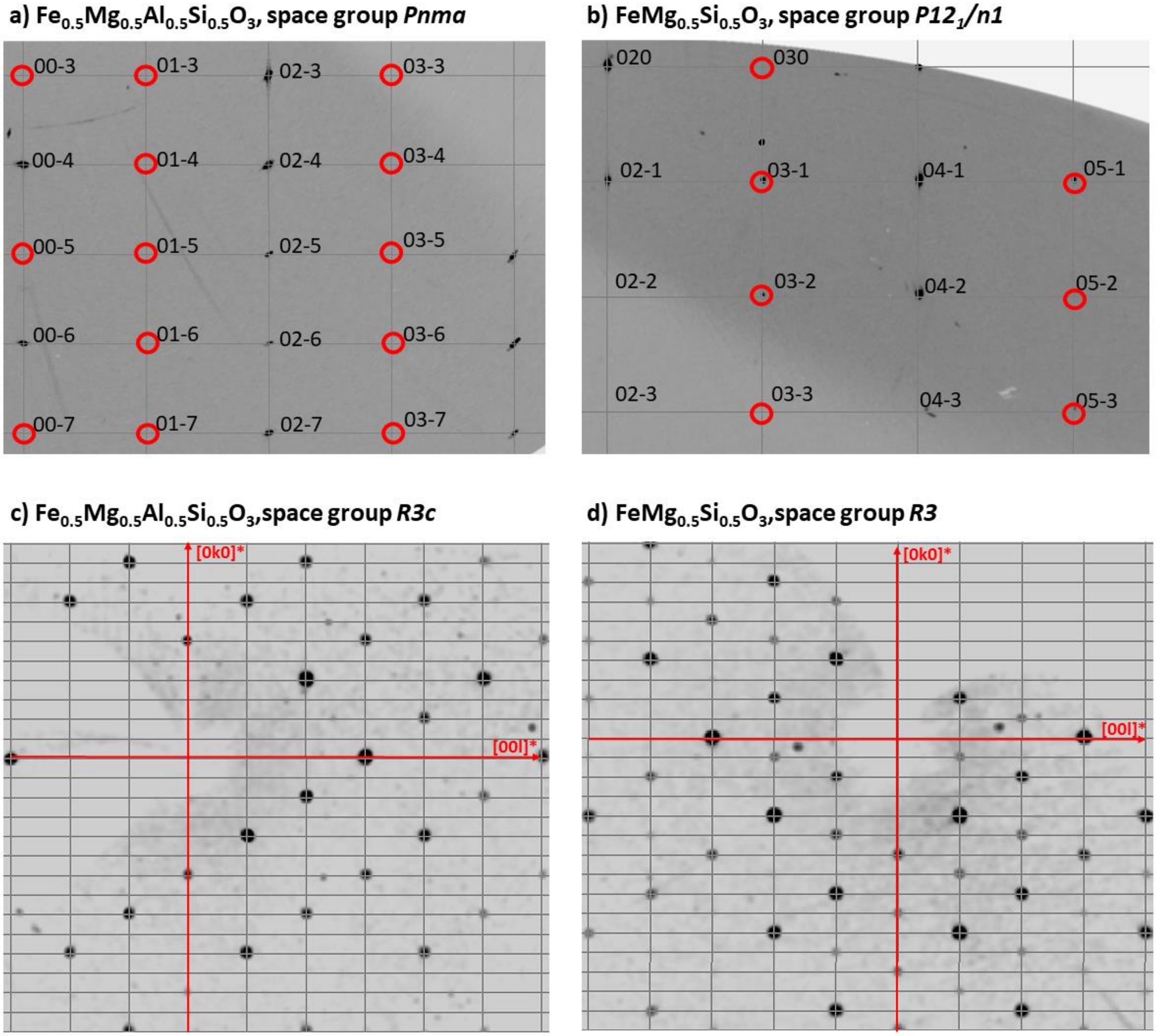

Supplementary Figure 2: Reciprocal planes reconstructed from CCD frames with the UNWARP procedure of CrysAlis.

Based on single-crystal XRD in a diamond anvil cell at high pressure:

(a) $Fe_{0.5}Mg_{0.5}Si_{0.5}Al_{0.5}O_3$ Red circles mark systematic absences that are characteristic for space group *Pnma*.

(b) $FeMg_{0.5}Si_{0.5}O_3$ double perovskite. One cannot see systematic absences characteristic for *Pnma* space group here (red circles).

Based on in-house single-crystal XRD at ambient pressure:

(c) Low-pressure $LiNbO_3$-type phase of FMAS sample, space group *R3c*.

(d) Low-pressure phase of FMS sample. Observation of *(0,0,3n)* reflections indicates absence of *c* glide plane, so space group is *R3*.

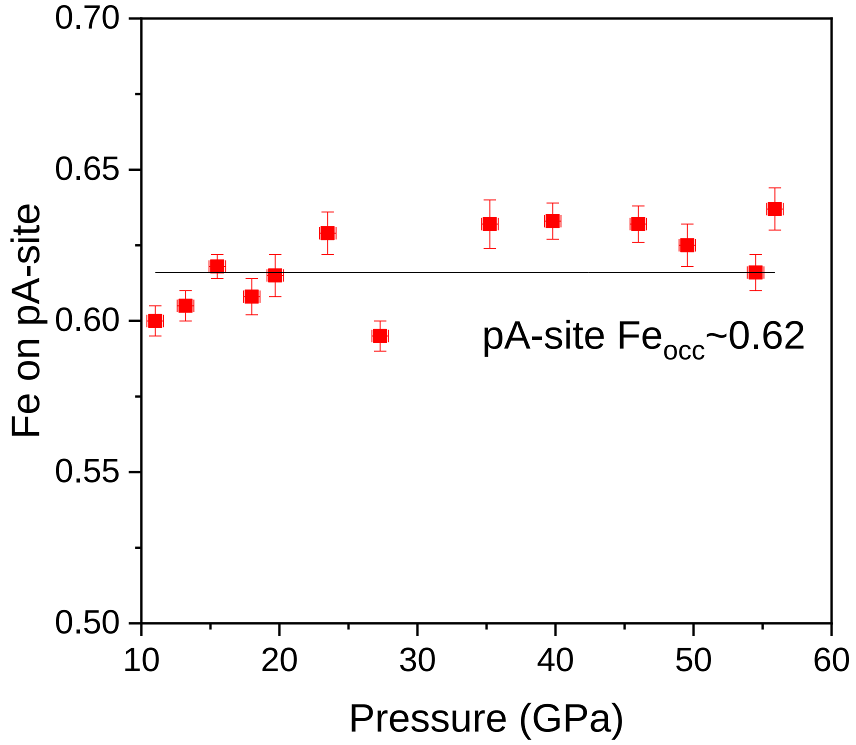

Supplementary Figure 3: Fe content on pA-site, occupied by Fe and Mg in $FeMg_{0.5}Si_{0.5}O_3$ double-perovskite at different pressures.

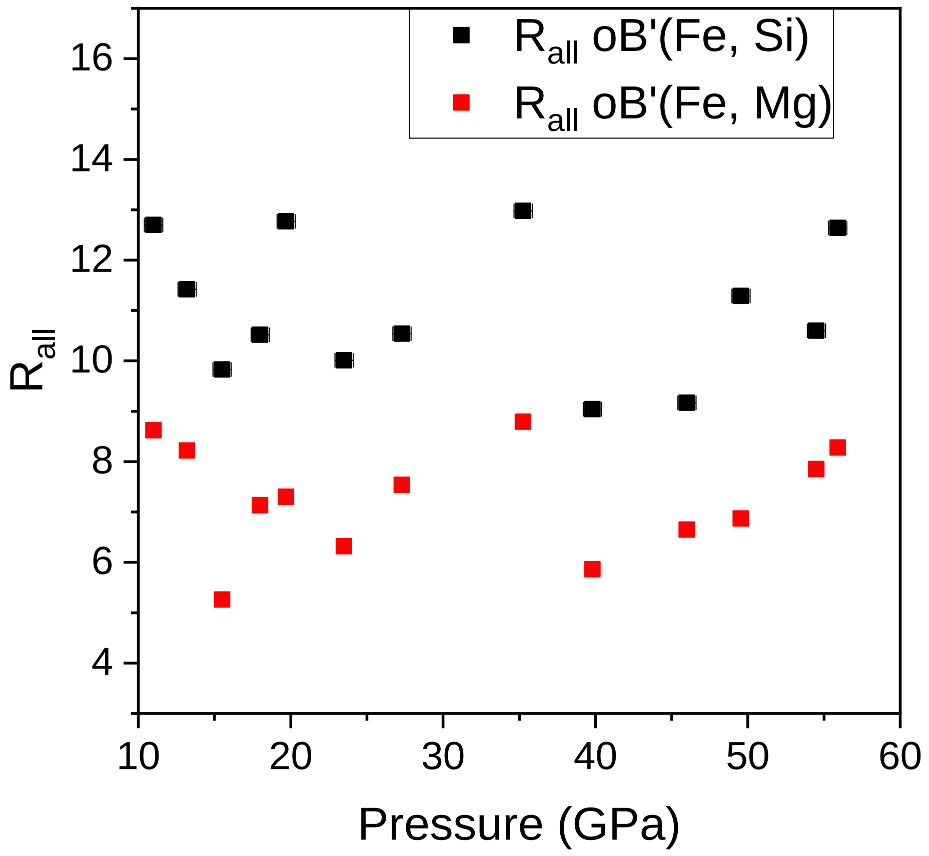

Supplementary Figure 4: R_{all} versus pressure for two double-perovskite structures with different oB'-site cation occupancies. Black squares marks oB'-site occupied by Fe and Si, red squares for oB'-site occupied by Fe and Mg. Detection of Mg on oB' site of double perovskite is not an artefact of data processing, because structure refinement without Mg leads to R_{all} increase at all pressure points.

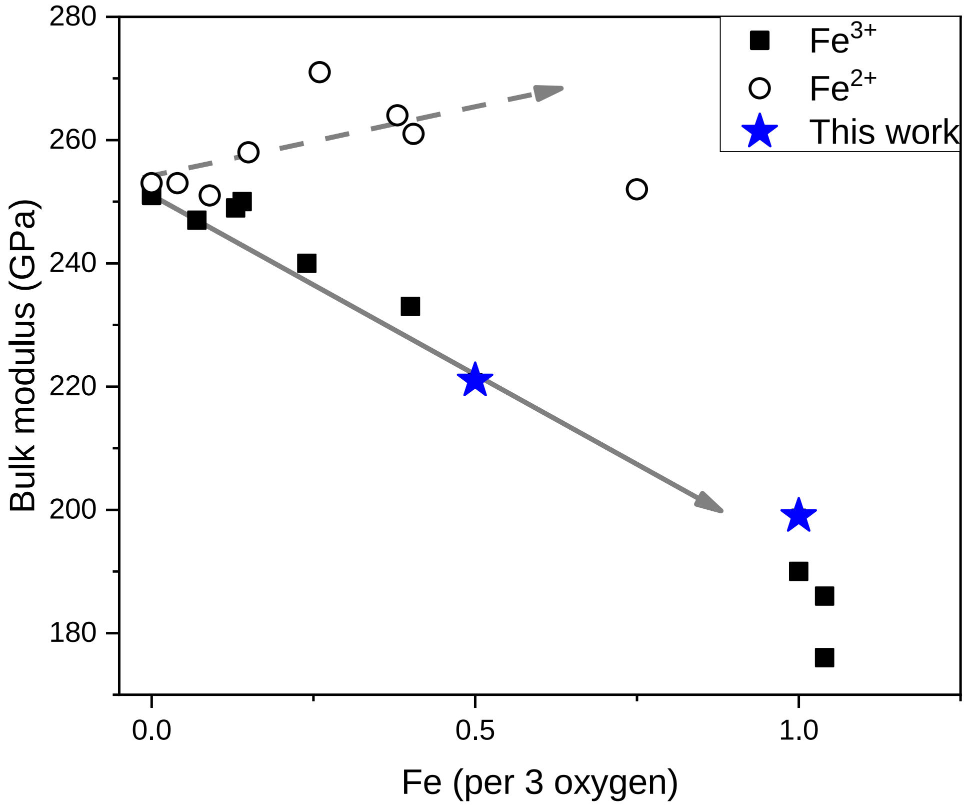

Supplementary Figure 5. Bulk modulus of various Fe-rich silicates reported previously(Lavina et al., 2010; Glazyrin, 2011; Ismailova et al., 2016; Liu et al., 2018; W and Vasiukov, 2018).

Sample	O	Mg	Fe	Si	Al
$Fe_{0.5}Mg_{0.5}Al_{0.5}Si_{0.5}O_3$	3.00(1)	0.49(1)	0.49(1)	0.51(1)	0.52(1)
$FeMg_{0.5}Si_{0.5}O_3$	2.98(4)	0.50(1)	1.00(2)	0.52(1)	-

Supplementary Table 1. Chemical compositions of $Fe_{0.5}Mg_{0.5}Al_{0.5}Si_{0.5}O_3$ and $FeMg_{0.5}Si_{0.5}O_3$ samples based on structure refinements averaged data points with standard deviation as an uncertainty. Cation proportions normalized for 5 atoms per formula unit. In the main text and for the constrains during structure refinement we use simplified compositions, $FeMg_{0.5}Si_{0.5}O_3$ for and $Fe_{0.5}Mg_{0.5}Si_{0.5}Al_{0.5}O_3$. The estimation of oxygen vacancies in $FeMg_{0.5}Si_{0.5}O_3$ sample is 0-2% atoms per formula unit from microprobe data. From charge balance together with Mossbauer spectroscopy, which showed approximately 16(4)% of Fe^{2+}, one can estimate that less than 1% of oxygen positions are vacant. From single crystal XRD at high pressure point of view this minor amount cannot be estimated, so we assumed no oxygen vacansies during structure solution and refinement.

Filename	FMS_m_18GPa	**FMS_m_19.7GPa***	FMS_m_15.5GPa	FMS_m_13.2GPa	FMS_m_11GPa
Chemical formula	$Fe_{0.96}Mg_{0.5}Si_{0.54}O_3$	$Fe_{0.96}Mg_{0.5}Si_{0.54}O_3$	$Fe_{0.96}Mg_{0.5}Si_{0.54}O_3$	$Fe_{0.96}Mg_{0.5}Si_{0.54}O_3$	$Fe_{0.96}Mg_{0.5}Si_{0.54}O_3$
Crystal system, space group	Monoclinic, $P2_1/n$	Monoclinic, $P2_1/n$	Monoclinic, $P2_1/n$	Monoclinic, $P2_1/n$	Monoclinic, $P2_1/n$
Temperature (K)<br>Pressure (GPa)	293<br>18.0(6)	293<br>19.7(6)	293<br>15.5(6)	293<br>13.2(6)	293<br>11.0(6)
a, *b*, *c* (Å)	4.7647 (2)<br>5.0139 (2)<br>7.0170 (9)	4.7474 (1)<br>5.0056 91)<br>7.0100 (9)	4.7764 (1)<br>5.0253 (2)<br>7.0536 (9)	4.7918 (1)<br>5.0346 (2)<br>7.0670 (9)	4.8036 (1)<br>5.0444 (2)<br>7.1000 (7)
β (°)	90.182 (7)	90.068 (6)	90.031 (3)	90.025 (9)	90.005 (4)
V (Å^3)	167.63 (3)	166.58 (3)	169.31 (2)	170.49 (2)	172.04 (2)
Z	4	4	4	4	4
F(000)	250	250	250	250	250
D_x (Mg m^{-3})	5.109	5.141	5.058	5.023	4.978
θ range (°) for cell measurement	3.0–20.3	3.4–19.8	3.4–19.7	3.4–19.6	5.0–19.9
μ (mm^{-1})	1.8	1.81	1.79	1.77	1.76
No. of measured, independent and observed reflections	331, 211, 197	370, 208, 196	358, 210, 196	381, 229, 205	385, 209, 199
R_{int}	0.02	0.025	0.023	0.051	0.029
$(\sin \theta/\lambda)_{max}$ (Å^{-1})	0.847	0.824	0.82	0.817	0.83
Range of *h*, *k*, *l*	*h* = -6→6, *k* = -7→6, *l* = -3→6	*h* = -7→7, *k* = -7→7, *l* = -6→5	*h* = -7→7, *k* = -7→6, *l* = -6→5	*h* = -7→7, *k* = -7→7, *l* = -5→6	*h* = -7→7, *k* = -7→7, *l* = -5→6
$R[F^2 > 2\sigma(F^2)]$, $wR(F^2)$, S	0.076, 0.092, 4.77	0.073, 0.189, 4.25	0.049, 0.076, 3.80	0.078, 0.095, 4.31	0.083, 0.113, 5.02
No. of reflections	211	196	210	229	209
No. of parameters	20	20	20	20	20
No. of restraints	0	0	0	0	0
No. of constraints	4	4	4	4	4

Supplementary table 2 Details of structure refinement of high-pressure $FeMg_{0.5}Si_{0.5}O_3$ double-perovskite. *CIF-file is available via CCDC, deposition number 2294967

Filename	FMS_m_23.5GPa	FMS_m_27.3GPa	FMS_m_35.2GPa	FMS_m_39.8GPa	FMS_m_45.6GPa
Chemical formula	$Fe_{0.96}Mg_{0.5}Si_{0.54}O_3$	$Fe_{0.96}Mg_{0.5}Si_{0.54}O_3$	$Fe_{0.96}Mg_{0.5}Si_{0.54}O_3$	$Fe_{0.96}Mg_{0.5}Si_{0.54}O_3$	$Fe_{0.96}Mg_{0.5}Si_{0.54}O_3$
Crystal system, space group	Monoclinic, $P2_1/n$	Monoclinic, $P2_1/n$	Monoclinic, $P2_1/n$	Monoclinic, $P2_1/n$	Monoclinic, $P2_1/n$
Temperature (K)<br>Pressure (GPa)	293<br>23.5(6)	293<br>27.3(6)	293<br>35.2(6)	293<br>39.8(6)	293<br>45.6(6)
a, *b*, *c* (Å)	4.7319(4)<br>4.9975(2)<br>6.965(4)	4.706(2)<br>4.9840(2)<br>6.9262(5)	4.6595(4)<br>4.9575(2)<br>6.8560(5)	4.631(2)<br>4.9372(2)<br>6.813(2)	4.586(3)<br>4.9085(2)<br>6.7436(5)
β (°)	89.93(3)	89.99(5)	89.93(4)	90.05(4)	89.98(7)
V ($Å^3$)	164.71(2)	162.5(1)	158.37(2)	155.78(8)	151.891)
Z	4	4	4	4	4
F(000)	250	250	250	250	250
D_x (Mg m^{-3})	5.199	5.271	5.407	5.497	5.64
θ range (°) for cell measurement	5.3–19.7	5.0–19.8	3.1–19.9	3.1–19.5	3.1–20
μ (mm^{-1})	1.84	1.86	1.91	1.94	1.99
No. of measured, independent and observed reflections	324, 162, 150	295, 262, 241	310, 273, 237	270, 244, 232	298, 298, 278
R_{int}	0.025	0.025	0.028	0.018	0.03
$(\sin \theta/\lambda)_{max}$ ($Å^{-1}$)	0.818	0.822	0.823	0.81	0.815
Range of *h*, *k*, *l*	*h* = -5→4, *k* = -7→7, *l* = -7→8	*h* = -4→5, *k* = -7→7, *l* = -8→7	*h* = -4→5, *k* = -7→7, *l* = -8→7	*h* = -4→4, *k* = -7→7, *l* = -8→7	*h* = -4→5, *k* = -7→7, *l* = -8→7
$R[F^2 > 2\sigma(F^2)]$, $wR(F^2)$, *S*	0.061, 0.087, 4.94	0.064, 0.152, 3.43	0.076, 0.089, 3.68	0.057, 0.080, 4.39	0.065, 0.092, 4.61
No. of reflections	162	262	273	244	298
No. of parameters	20	20	20	20	20
No. of restraints	0	0	0	0	0
No. of restraints	4	4	4	4	4

Supplementary table 2 (continued) Details of structure refinement of high-pressure $FeMg_{0.5}Si_{0.5}O_3$ double-perovskite

Filename	FMS_m_49.5GPa	FMS_m_54.5GPa	FMS_m_55.9GPa	**FMS_t_0GPa***
Chemical formula	$Fe_{0.96}Mg_{0.5}Si_{0.54}O_3$	$Fe_{0.96}Mg_{0.5}Si_{0.54}O_3$	$Fe_{0.96}Mg_{0.5}Si_{0.54}O_3$	$FeMg_{0.5}Si_{0.5}O_3$
Crystal system, space group	Monoclinic, $P2_1/n$	Monoclinic, $P2_1/n$	Monoclinic, $P2_1/n$	Trigonal, *R*3
Temperature (K)<br>Pressure (GPa)	293<br>49.5(6)	293<br>54.5(6)	293<br>55.9(6)	293<br>0.0001
a, *b*, *c* (Å)	4.558(3)<br>4.8875(5)<br>6.689(3)	4.526(6)<br>4.8779(7)<br>6.647(8)	4.527(3)<br>4.8701(3)<br>6.641(3)	4.9406(7)<br><br>13.319(2)
β (°)	90.17(6)	89.7(1)	90.15(6)	
V ($Å^3$)	149.0(1)	146.8(3)	146.4(1)	281.74(7)
Z	4	4	4	6
F(000)	250	250	250	375
D_x (Mg m^{-3})	5.746	5.834	5.848	4.565
θ range (°) for cell measurement	3.0–19.7	3.0–18.6	4.0–18.9	3.9–28.6
μ (mm^{-1})	2.03	2.06	2.06	4.07
No. of measured, independent and observed reflections	324, 151, 132	318, 273, 217	307, 271, 227	1076, 518, 446
R_{int}	0.018	0.045	0.069	0.038
$(\sin \theta/\lambda)_{max}$ ($Å^{-1}$)	0.819	0.821	0.824	0.846
Range of *h*, *k*, *l*	*h* = -5→4, *k* = -7→7, *l* = -7→8	*h* = -5→4, *k* = -7→7, *l* = -7→8	*h* = -5→4, *k* = -7→7, *l* = -7→8	*h* = -8→7, *k* = -6→8, *l* = -16→22
$R[F^2 > 2\sigma(F^2)]$, $wR(F^2)$, *S*	0.065, 0.073, 3.95	0.068, 0.080, 2.80	0.079, 0.168, 3.46	0.054, 0.061, 3.87
No. of reflections	151	273	271	446
No. of parameters	20	20	20	33
No. of restraints	0	0	0	0
No. of restraints	4	4	4	6

Supplementary table 2 (continued) Details of structure refinement of high-pressure $FeMg_{0.5}Si_{0.5}O_3$ double-perovskite and its' low-pressure polymorph. *CIF-file is available via CCDC, deposition number 2294965

Filename	FMAS_t_0GPa	FMAS_t_3.2GPa	**FMAS_t_5.5GPa***	FMAS_t_5GPa	FMAS_t_8.8GPa
Chemical formula	$Fe_{0.5}Mg_{0.5}Si_{0.5}Al_{0.5}O_3$	$Fe_{0.5}Mg_{0.5}Si_{0.5}Al_{0.5}O_3$	$Fe_{0.5}Mg_{0.5}Si_{0.5}Al_{0.5}O_3$	$Fe_{0.5}Mg_{0.5}Si_{0.5}Al_{0.5}O_3$	$Fe_{0.5}Mg_{0.5}Si_{0.5}Al_{0.5}O_3$
Crystal system, space group	Trigonal, *R*3*c*	Trigonal, *R*3*c*	Trigonal, *R*3*c*	Trigonal, *R*3*c*	Trigonal, *R*3*c*
Temperature (K)<br>Pressure (GPa)	293<br>0.0001	293<br>3.2(9)	293<br>5.5(9)	293<br>5.0(5)	293<br>8.8(5)
a, *b*, *c* (Å)	4.8790 (1)<br>12.9112 (1)	4.8632 (8)<br>12.8060 (18)	4.8475 (8)<br>12.7880 (18)	4.8454 (8)<br>12.7870 (18)	4.8263 (8)<br>12.7063 (18)
β (°)	266.17 (1)	262.29 (7)	260.24 (7)	259.99 (7)	256.32 (7)
V (Å^3)	6	6	6	6	6
Z	339	339	339	339	339
F(000)	4.233	4.327	4.366	4.314	4.494
D_x (Mg m^{-3})	4.6–28.5	3.3–16.8	3.4–19.1	7.5–19.8	6.0–19.9
θ range (°) for cell measurement	2.24	0.96	0.97	0.9	1.06
μ (mm^{-1})	952, 787, 719	177, 177, 174	166, 166, 158	183, 143, 143	184, 184, 178
No. of measured, independent and observed reflections	0.04	0.055	0.062	0.036	0.043
R_{int}	0.857	0.855	0.796	0.836	0.84
(sin θ/λ)$_{max}$ (Å^{-1})	h = -6→8, k = -7→6, l = -20→12	h = -7→7, k = -7→8, l = -9→9	h = -7→7, k = -7→7, l = -8→9	h = -6→7, k = -5→3, l = -19→14	h = -5→3, k = -6→7, l = -15→19
Range of h, k, l	0.050, 0.054, 1.88	0.050, 0.064, 2.10	0.065, 0.075, 3.77	0.074, 0.096, 4.89	0.073, 0.120, 5.24
$R[F^2 > 2\sigma(F^2)]$, $wR(F^2)$, S	787	177	166	143	178
No. of reflections	11	11	11	11	11
No. of parameters	0	0	0	0	0
No. of parameters	6	6	6	6	6

Supplementary table 3 Details of structure refinement of $Fe_{0.5}Mg_{0.5}Si_{0.5}Al_{0.5}O_3$. *CIF-file is available via CCDC, deposition number 2294968

Filename	**FMAS_o_11.5GPa***	FMAS_o_15GPa	FMAS_o_18.3GPa	FMAS_o_31.6GPa	FMAS_o_35.7GPa
Chemical formula	$Fe_{0.5}Mg_{0.5}Si_{0.5}Al_{0.5}O_3$	$Fe_{0.5}Mg_{0.5}Si_{0.5}Al_{0.5}O_3$	$Fe_{0.5}Mg_{0.5}Si_{0.5}Al_{0.5}O_3$	$Fe_{0.5}Mg_{0.5}Si_{0.5}Al_{0.5}O_3$	$Fe_{0.5}Mg_{0.5}Si_{0.5}Al_{0.5}O_3$
Crystal system, space group	Orthorhombic, *Pnma*	Orthorhombic, *Pnma*	Orthorhombic, *Pnma*	Orthorhombic, *Pnma*	Orthorhombic, *Pnma*
Temperature (K)<br>Pressure (GPa)	293<br>11.5(6)	293<br>15.0(6)	293<br>18.3(6)	293<br>31.6(6)	293<br>35.7(6)
a, *b*, *c* (Å)	4.9613 (13)<br>6.9397 (15)<br>4.7359 (6)	4.9429 (13)<br>6.9027 (15)<br>4.7108 (6)	4.9196 (13)<br>6.8905 (15)<br>4.6922 (6)	4.8714 (13)<br>6.7794 (15)<br>4.6283 (6)	4.8524 (13)<br>6.7378 (15)<br>4.6001 (6)
β (°)	163.06 (6)	160.73 (6)	159.06 (6)	152.85 (6)	150.40 (6)
V (Å^3)	4	4	4	4	4
Z	226	226	226	226	226
F(000)	4.613	4.68	4.758	4.917	5.001
D_x (Mg m^{-3})	3.8–20.2	3.9–20.7	3.0–20.5	3.1–20.4	3.1–20.4
θ range (°) for cell measurement	0.99	1	1.05	1.05	1.07
μ (mm^{-1})	303, 121, 113	392, 392, 289	308, 106, 94	373, 373, 332	347, 347, 263
No. of measured, independent and observed reflections	0.03	0.053	0.049	0.033	0.037
R_{int}	0.841	0.859	0.862	0.85	0.853
(sin θ/λ)$_{max}$ (Å^{-1})	h = -5→3, k = -10→8, l = -5→5	h = -6→4, k = -10→9, l = -5→5	h = -6→3, k = -9→10, l = -5→5	h = -4→5, k = -9→9, l = -6→7	h = -5→4, k = -9→9, l = -6→7
Range of h, k, l	0.084, 0.102, 4.35	0.087, 0.096, 4.04	0.073, 0.089, 3.37	0.055, 0.079, 3.59	0.057, 0.076, 3.53
$R[F^2 > 2\sigma(F^2)]$, $wR(F^2)$, S	121	289	106	332	263
No. of reflections	12	12	12	12	12
No. of parameters	0	0	0	0	0
No. of restraints	4	4	4	4	4

Supplementary table 3 (continued) Details of structure refinement of $Fe_{0.5}Mg_{0.5}Si_{0.5}Al_{0.5}O_3$. *CIF-file is available via CCDC, deposition number 2294966

Filename	FMAS_o_40GPa	FMAS_o_47.2GPa	FMAS_o_55.1GPa	FMAS_o_7.5GPa	FMAS_o_8.6GPa
Chemical formula	$Fe_{0.5}Mg_{0.5}Si_{0.5}Al_{0.5}O_3$	$Fe_{0.5}Mg_{0.5}Si_{0.5}Al_{0.5}O_3$	$Fe_{0.5}Mg_{0.5}Si_{0.5}Al_{0.5}O_3$	$Fe_{0.5}Mg_{0.5}Si_{0.5}Al_{0.5}O_3$	$Fe_{0.5}Mg_{0.5}Si_{0.5}Al_{0.5}O_3$
Crystal system, space group	Orthorhombic, *Pnma*	Orthorhombic, *Pnma*	Orthorhombic, *Pnma*	Orthorhombic, *Pnma*	Orthorhombic, *Pnma*
Temperature (K)<br>Pressure (GPa)	293<br>40.0(6)	293<br>47.2(6)	293<br>55.1(6)	293<br>7.5(6)	293<br>8.6(6)
a, *b*, *c* (Å)	4.8443 (13)<br>6.7189 (15)<br>4.5876 (6)	4.8174 (13)<br>6.6662 (15)<br>4.5535 (6)	4.8007 (13)<br>6.6260 (15)<br>4.5223 (6)	4.9720 (13)<br>6.9850 (15)<br>4.7880 (6)	4.9860 (13)<br>7.0020 (15)<br>4.7759 (6)
β (°)	149.32 (6)	146.23 (5)	143.85 (5)	166.28 (6)	166.74 (6)
V (Å^3)	4	4	4	4	4
Z	226	226	226	226	226
F(000)	5.039	5.144	5.229	4.523	4.511
D_x (Mg m^{-3})	3.5–19.8	6.3–19.6	3.2–19.7	3.0–18.7	3–18.7
θ range (°) for cell measurement	1.08	1.1	1.12	0.97	0.97
μ (mm^{-1})	345, 345, 287	318, 318, 222	293, 293, 194	282, 154, 111	288, 150, 141
No. of measured, independent and observed reflections	0.027	0.054	0.04	0.055	0.034
R_{int}	0.855	0.859	0.825	0.825	0.852
(sin θ/λ)$_{max}$ (Å^{-1})	*h* = -5→4, *k* = -9→9, *l* = -6→7	*h* = -4→5, *k* = -8→9, *l* = -5→5	*h* = -3→5, *k* = -8→9, *l* = -5→5	*h* = -5→3, *k* = -9→10, *l* = -6→5	*h* = -3→5, *k* = -10→8, *l* = -6→6
Range of *h*, *k*, *l*	0.056, 0.076, 3.39	0.065, 0.096, 4.87	0.057, 0.074, 3.57	0.057, 0.063, 1.87	0.077, 0.184, 3.52
$R[F^2 > 2\sigma(F^2)]$, $wR(F^2)$, S	287	222	194	111	150
No. of reflections	12	12	12	12	12
No. of parameters	0	0	0	0	0
No. of restraints	4	4	4	4	4

Supplementary table 3 (continued) Details of structure refinement of $Fe_{0.5}Mg_{0.5}Si_{0.5}Al_{0.5}O_3$

Filename	FMAS_o_10.8GPa	FMAS_o_13.6GPa	FMAS_o_17.5GPa	FMAS_o_19.7GPa	FMAS_o_15.2GPa
Chemical formula	$Fe_{0.5}Mg_{0.5}Si_{0.5}Al_{0.5}O_3$	$Fe_{0.5}Mg_{0.5}Si_{0.5}Al_{0.5}O_3$	$Fe_{0.5}Mg_{0.5}Si_{0.5}Al_{0.5}O_3$	$Fe_{0.5}Mg_{0.5}Si_{0.5}Al_{0.5}O_3$	$Fe_{0.5}Mg_{0.5}Si_{0.5}Al_{0.5}O_3$
Crystal system, space group	Orthorhombic, *Pnma*	Orthorhombic, *Pnma*	Orthorhombic, *Pnma*	Orthorhombic, *Pnma*	Orthorhombic, *Pnma*
Temperature (K)<br>Pressure (GPa)	293<br>10.8(6)	293<br>13.6(6)	293<br>17.5(6)	293<br>19.7(6)	293<br>15.2(6)
a, *b*, *c* (Å)	4.9612 (13)<br>6.9577 (15)<br>4.7474 (6)	4.9488 (13)<br>6.9119 (15)<br>4.7309 (6)	4.9357 (13)<br>6.8908 (15)<br>4.7124 (6)	4.9273 (13)<br>6.8810 (15)<br>4.6974 (6)	4.9457 (13)<br>6.9127 (15)<br>4.7194 (6)
β (°)	163.87 (6)	161.82 (6)	160.27 (6)	159.26 (6)	161.35 (6)
V (Å^3)	4	4	4	4	4
Z	226	226	226	226	226
F(000)	4.59	4.648	4.693	4.723	4.662
D_x (Mg m^{-3})	3.4–20.4	3.0–20.7	3.0–19.3	3.9–20.7	3.9–20.6
θ range (°) for cell measurement	0.99	1	1.01	1.01	1
μ (mm^{-1})	337, 145, 134	350, 163, 152	339, 149, 139	284, 160, 150	279, 139, 112
No. of measured, independent and observed reflections	0.025	0.02	0.019	0.014	0.054
R_{int}	0.841	0.861	0.836	0.859	0.855
(sin θ/λ)$_{max}$ (Å^{-1})	*h* = -3→5, *k* = -10→10, *l* = -6→6	*h* = -4→5, *k* = -9→10, *l* = -6→6	*h* = -5→4, *k* = -10→9, *l* = -6→6	*h* = -4→5, *k* = -10→8, *l* = -7→6	*h* = -4→5, *k* = -10→8, *l* = -7→6
Range of *h*, *k*, *l*	0.081, 0.095, 4.84	0.078, 0.098, 5.60	0.070, 0.094, 5.17	0.064, 0.080, 4.76	0.098, 0.100, 3.42
$R[F^2 > 2\sigma(F^2)]$, $wR(F^2)$, S	134	152	139	150	112
No. of reflections	12	12	12	12	12
No. of parameters	0	0	0	0	0
No. of restraints	4	4	4	4	4

Supplementary table 3 (continued) Details of structure refinement of $Fe_{0.5}Mg_{0.5}Si_{0.5}Al_{0.5}O_3$

Filename	FMAS_o_13.5GPa	FMAS_o_11GPa	FMAS_o_60GPa
Chemical formula	$Fe_{0.5}Mg_{0.5}Si_{0.5}Al_{0.5}O_3$	$Fe_{0.5}Mg_{0.5}Si_{0.5}Al_{0.5}O_3$	$Fe_{0.5}Mg_{0.5}Si_{0.5}Al_{0.5}O_3$
Crystal system, space group	Orthorhombic, *Pnma*	Orthorhombic, *Pnma*	Orthorhombic, *Pnma*
Temperature (K)<br>Pressure (GPa)	293<br>13.5(6)	293<br>11.0(6)	293<br>60.0(6)
a, *b*, *c* (Å)	4.9540 (13)<br>6.9287 (15)<br>4.7335 (6)	4.9641 (13)<br>6.9542 (15)<br>4.7459 (6)	4.7835 (13)<br>6.6033 (15)<br>4.5020 (6)
β (°)	162.48 (6)	163.83 (6)	142.20 (5)
V (Å^3)	4	4	4
Z	226	226	226
F(000)	4.629	4.591	5.297
D_x (Mg m^{-3})	3.0–19.1	3.8–19.0	5.8–19.6
θ range (°) for cell measurement	1	0.99	1.15
μ (mm^{-1})	288, 148, 112	290, 148, 113	306, 237, 232
No. of measured, independent and observed reflections	0.035	0.037	0.042
R_{int}	0.794	0.791	$□_{max}$ = 19.9, $□_{min}$ = 3.2
$(\sin \theta/\lambda)_{max}$ (Å^{-1})	h = -4→5, k = -9→8, l = -7→6	h = -4→5, k = -9→8, l = -7→6	h = -4→5, k = -8→9, l = -5→5
Range of *h*, *k*, *l*	0.075, 0.088, 3.11	0.094, 0.093, 3.25	0.069, 0.107, 4.89
$R[F^2 > 2\sigma(F^2)]$, $wR(F^2)$, S	112	113	237
No. of reflections	12	12	12
No. of parameters	0	0	0
No. of restraints	4	4	4

Supplementary table 3 (continued) Details of structure refinement of $Fe_{0.5}Mg_{0.5}Si_{0.5}Al_{0.5}O_3$

Supplementary Table 4: Mössbauer parameters summary.

**$FeMg_{0.5}Si_{0.5}O_3$ double-perovskite**										
P (GPa)	*CS*	*error*	*FWHM*	*error*	*Int*	*error*	*QS*	*error*	*a12*	*error*
**Fe^{3+} on pA-site**										
14.9	0.373	0.045	0.492	0.198	81.785	25.305	1.088	0.099	0.376	0.064
25.6	0.405	0.030	0.521	0.099	88.652	17.649	1.098	0.052	0.389	0.031
49.5	0.407	0.025	0.433	0.054	70.047	9.338	1.168	0.061	0.339	0.026
62	0.332	0.020	0.414	0.073	83.432	10.232	1.278	0.021	0.425	0.017
78	0.297	0.014	0.477	0.054	88.094	7.132	1.326	0.021	0.379	0.015
95	0.285	0.011	0.527	0.039	85.726	5.242	1.308	0.014	0.432	0.010
**Fe^{3+} on oB'-site**										
14.9	0.165	0.093	0.132	3.370	18.215	25.305	0.402	0.164	0.376	0.064
25.6	0.082	0.060	0.237	0.290	11.348	17.649	0.791	0.158	0.389	0.031
49.5	0.059	0.044	0.268	0.278	29.953	9.338	0.784	0.097	0.339	0.026
62	-0.152	0.078	0.233	0.693	16.568	10.232	1.215	0.083	0.425	0.017
78	-0.238	0.057	0.174	0.991	11.906	7.132	1.206	0.082	0.379	0.015
95	-0.266	0.028	0.173	0.216	14.274	5.242	1.280	0.037	0.432	0.010
**$Fe_{0.5}Mg_{0.5}Al_{0.5}Si_{0.5}O_3$ perovskite**										
P (GPa)	*CS*	*error*	*FWHM*	*error*	*Int*	*error*	*QS*	*error*	*a12*	*error*
**Fe^{3+} on pA-site**										
19	0.416	0.013	0.639	0.033	85.803	12.645	1.130	0.032	0.486	0.010
32	0.387	0.013	0.677	0.036	82.110	13.659	1.196	0.034	0.488	0.008
44	0.371	0.014	0.717	0.045	81.037	16.521	1.279	0.040	0.476	0.010
53	0.352	0.012	0.745	0.042	83.363	13.352	1.322	0.032	0.482	0.009
61	0.348	0.013	0.705	0.050	78.146	15.033	1.313	0.034	0.483	0.010
62	0.355	0.008	0.579	0.052	87.041	4.771	1.302	0.014	0.482	0.008
**Fe^{2+} on pA-site**										
19	0.813	0.118	0.803	0.253	14.197	12.645	2.552	0.155	0.486	0.010
32	0.767	0.144	1.212	0.366	17.890	13.659	2.677	0.256	0.488	0.008
44	0.905	0.198	1.310	0.447	18.963	16.521	2.967	0.264	0.476	0.010
53	0.783	0.151	1.260	0.350	16.637	13.352	3.167	0.215	0.482	0.009
61	0.710	0.127	1.401	0.365	21.854	15.033	3.109	0.271	0.483	0.010
62	1.027	0.040	0.679	0.125	12.959	4.771	3.725	0.071	0.482	0.008

References

Glazyrin, K. (2011). Iron in oxides , silicates and alloys under extreme pressure-temperature conditions.

Ismailova, L., Bykova, E., Bykov, M., Cerantola, V., McCammon, C., Boffa Ballaran, T., et al. (2016). Stability of Fe, Al-bearing bridgmanite in the lower mantle and synthesis of pure Fe-bridgmanite. *Sci Adv* 2, e1600427. doi: 10.1126/sciadv.1600427.

Lavina, B., Dera, P., Downs, R. T., Yang, W., Sinogeikin, S., Meng, Y., et al. (2010). Structure of siderite FeCO3 to 56 GPa and hysteresis of its spin-pairing transition. *Phys Rev B* 82, 064110. doi: 10.1103/PhysRevB.82.064110.

Liu, J., Dorfman, S. M., Zhu, F., Li, J., Wang, Y., Zhang, D., et al. (2018). Valence and spin states of iron are invisible in Earth's lower mantle. *Nat Commun* 9, 1–9. doi: 10.1038/s41467-018-03671-5.

W, E. Der, and Vasiukov, D. (2018). Iron electronic states in minerals of the Earth ' s mantle eingereicht an der Bayreuther Graduiertenschule f ¨ ur Mathematik und Naturwissenschaften (BayNAT) submitted to the Bayreuth Graduate School of Mathematics and Natural Sciences (BayNAT).
